# Immunohistochemical localization of NGF, BDNF, and their receptors in a normal and AMD-like rat retina

**DOI:** 10.1186/s12920-019-0493-8

**Published:** 2019-03-13

**Authors:** Darya V. Telegina, Nataliya G. Kolosova, Oyuna S. Kozhevnikova

**Affiliations:** 1 Institute of Cytology and Genetics, SB RAS, Novosibirsk, Russia; 2 N. N. Vorozhtsov Novosibirsk Institute of Organic Chemistry, SB RAS, Novosibirsk, Russia

**Keywords:** Age-related macular degeneration, Retinopathy, Retina, NGF, BDNF, OXYS rats

## Abstract

**Background:**

Age-related macular degeneration (AMD) is a major cause of blindness in developed countries, and the molecular pathogenesis of AMD is poorly understood. A large body of evidence has corroborated the key role of neurotrophins in development, proliferation, differentiation, and survival of retinal cells. Neurotrophin deprivation has been proposed to contribute to retinal-cell death associated with neurodegenerative diseases. Little is known about the expression of the immature form of neurotrophins (proneurotrophins) and their mature form [e.g., nerve growth factor (proNGF and mNGF) and brain-derived neurotrophic factor (proBDNF and mBDNF)] in the retina during physiological aging and against the background of AMD. In addition, cell-specific localization of proteins NGF and BDNF in the retina during AMD development is not clear. Here, we evaluated contributions of the age-related alterations in the neurotrophin system to the development of AMD-like retinopathy in OXYS rats.

**Methods:**

Male OXYS rats at preclinical (20 days), early (3 months), and late (18 months) stages of the disease and age-matched male Wistar rats (as controls) were used. We performed immunohistochemical localization of NGF, BDNF, and their receptors TrkA, TrkB, and p75NTR by fluorescence microscopy in retinal sections from OXYS and Wistar rats.

**Results:**

We found increased NGF staining in Muller cells in 18-month-old OXYS rats (progressive stage of retinopathy). In contrast, we observed only subtle changes in the labeling of mature BDNF (mBDNF) and TrkB during the development of AMD-like retinopathy in OXYS rats. Using colocalization with vimentin and NeuN, we detected a difference in the cell type–specific localization of mBDNF between OXYS and Wistar rats. We showed that the mBDNF protein was located in Muller cells in OXYS rats, whereas in the Wistar retina, mBDNF immunoreactivity was detected in Muller cells and ganglion cells. During the development of AMD-like retinopathy, proBDNF dominated over mBDNF during increasing cell loss in the OXYS retina.

**Conclusions:**

These data indicate that alterations in the balance of neurotrophic factors in the retina are involved in the development of AMD-like retinopathy in OXYS rats and confirm their participation in the pathogenesis of AMD in humans.

## Background

Age-related macular degeneration (AMD) is a complex neurodegenerative disease resulting in a loss of central vision in the elderly. Development of AMD is based on age-associated changes of retinal pigment epithelium (RPE), Bruch’s membrane, and choriocapillaris, but the exact mechanisms initiating the transition from typical age-related changes to the pathological process remain poorly understood. AMD is characterized by a progressive loss of neurons and photoreceptors thus leading to blindness. Retinal-cell death is controlled by complex mechanisms, which are unclear at present. This state of affairs hinders the development of effective treatments of AMD [1].

Neurotrophins like nerve growth factor (NGF) and brain-derived neurotrophic factor (BDNF) are known to play an important role in aging and development of neurodegenerative diseases such as Alzheimer’s disease and AMD [2, 3]. As far as we know, the involvement of neurotrophins in the pathophysiology of AMD has not been articulated in the literature. Mature NGF and BDNF (mNGF and mBDNF) are derived from their precursors (proNGF and proBDNF) after proteolytic cleavage in the extracellular space. The mature and precursor forms have opposite effects on cells. mNGF is the preferred ligand for a tyrosine kinase receptor called tropomyosin-receptor kinase A (TrkA), and mBDNF for TrkB. Binding of mature neurotrophins to receptors Trk is known to regulate the growth, survival, differentiation, function, and plasticity of neuronal cells. ProNGF and proBDNF bind to both p75^NTR^ and coreceptor sortilin with high affinity, thereby increasing apoptosis [3]. The effects of NGF and BDNF on target cells depend on the ratio of the mature form to precursor form, and also on the type of receptors codistributed on the cell surface. Nevertheless, few studies have been able to distinguish the mature from precursor form of NGF and BDNF owing to limitations of specific antibodies. The retina is a part of the central nervous system (CNS), and although the literature regarding the retinal expression of NGF and BDNF is vast, little is known about the differences in expression of neurotrophins between a normal aging retina and AMD-affected retina. Besides, the exact localization of proteins NGF and BDNF in the retinal cells involved in the process of AMD development and their functional significance are not clear [4].

There is evidence that a suitable experimental model of AMD is senescence-accelerated OXYS rats, which spontaneously develop a phenotype similar to human age-related disorders including AMD-like retinopathy [5–11]. Retinopathy that develops in OXYS rats already at a young age corresponds (in terms of clinical manifestations and morphological characteristics) to the dry atrophic form of AMD in humans. Nonetheless, neovascularization develops in some (~ 10–20%) of these rats with age. The clinical signs of AMD-like retinopathy appear by the age of 3 months in 100% of OXYS rats against the background of a reduction in the transverse area of the RPE, impairment of choroidal microcirculation, and retinal thinning [7]. The progression of these abnormalities in OXYS rats until the age of 10 months is accompanied by a significant reduction in thickness of the photoreceptor cell layer and a decrease in the number of photoreceptor cell nuclei of the outer nuclear layer, especially in the central part of the retina [12]. Significant pathological changes in the RPE manifest themselves as excessive accumulation of lipofuscin and amyloid in the RPE regions [6], disturbances in the morphology of the RPE sheet, including an increase in the proportion of multinucleated cells, hypertrophy, distortion of cell shape, and reactive gliosis [10].

We hypothesized that neurotrophic factors are involved in the neuronal retinal degeneration in OXYS rats. Therefore, the aim of this study was to assess the age-related changes in cell type–specific expression of mNGF, mBDNF, proBDNF and their receptors in the retina of OXYS and control Wistar rats and contributions of neurotrophic factors to the development of AMD-like pathology in OXYS rats, including the preclinical stage.

## Materials and methods

### Animals

Male senescence-accelerated OXYS rats at preclinical (age 20 days), early (3 months), and late (18 months) stages of the disease (four per each age group) and age-matched male Wistar rats (as controls) were obtained from the Breeding Experimental Animal Laboratory of the Institute of Cytology and Genetics, the Siberian Branch of the Russian Academy of Sciences (Novosibirsk, Russia). The experiments were conducted at the Center for Genetic Resources of Laboratory Animals at the Institute of Cytology and Genetics, Siberian Branch, Russian Academy of Sciences (RFMEFI61914X0005 and RFMEFI61914X0010). The OXYS rat strain was derived from the Wistar rat strain at the Institute of Cytology and Genetics as described earlier [13] and was registered in the Rat Genome Database (http://rgd.mcw.edu/). At this point, we have the 109th generation of OXYS rats with spontaneously developing accelerated senescence syndrome (including AMD-like retinopathy) inherited in a linked manner.

### Immunofluorescent staining

This procedure was performed by a standard indirect method as described previously [10, 14]. The eyes were removed and fixed in fresh 4% paraformaldehyde in PBS for 2 h, washed three times in PBS, and then cryoprotected in graded sucrose solutions (10, 20, and 30%). Posterior eyecups were embedded in Killik (Bio-Optica), frozen, and stored at − 80 °C. Tissue slices (10 μm thick) were prepared on a Microm HM-505 N cryostat at − 20 °C, transferred onto Polysine® glass slides (Menzel-Glaser), and stored at − 20 °C. The slices were incubated for 1 h in 5% BSA with 0.1% Triton X-100 in PBS, followed by overnight incubation at 4 °C with the primary antibodies to NGF (1:100; ab6199, Abcam), to TrkA (1250; ab72029, Abcam), to proBDNF (1:100; ab72440, Abcam), to mBDNF (1:250; GF35L-100 UG, Millipore), to TrkB (1:100; ab18987, Abcam), to p75^NTR^ (1:100; ab93934, Abcam), to NeuN (1:200; ab177487, Abcam), and to vimentin (1:200; ab24525, Abcam). After incubation with the respective secondary antibodies diluted 1:200, the slices were coverslipped with the Fluoroshield mounting medium containing 4*′*,6-diamidino-2-phenylindole (DAPI; Abcam) and examined under an Axioplan 2 microscope (Zeiss). The negative-control samples with the omitted primary antibody emitted only a minimal autofluorescent signal. At least four tissue slices (technical replicates) were analyzed per animal. For each image acquisition, all imaging parameters were the same.

## Results

### mNGF and TrkA expression

**Fig. 1:**
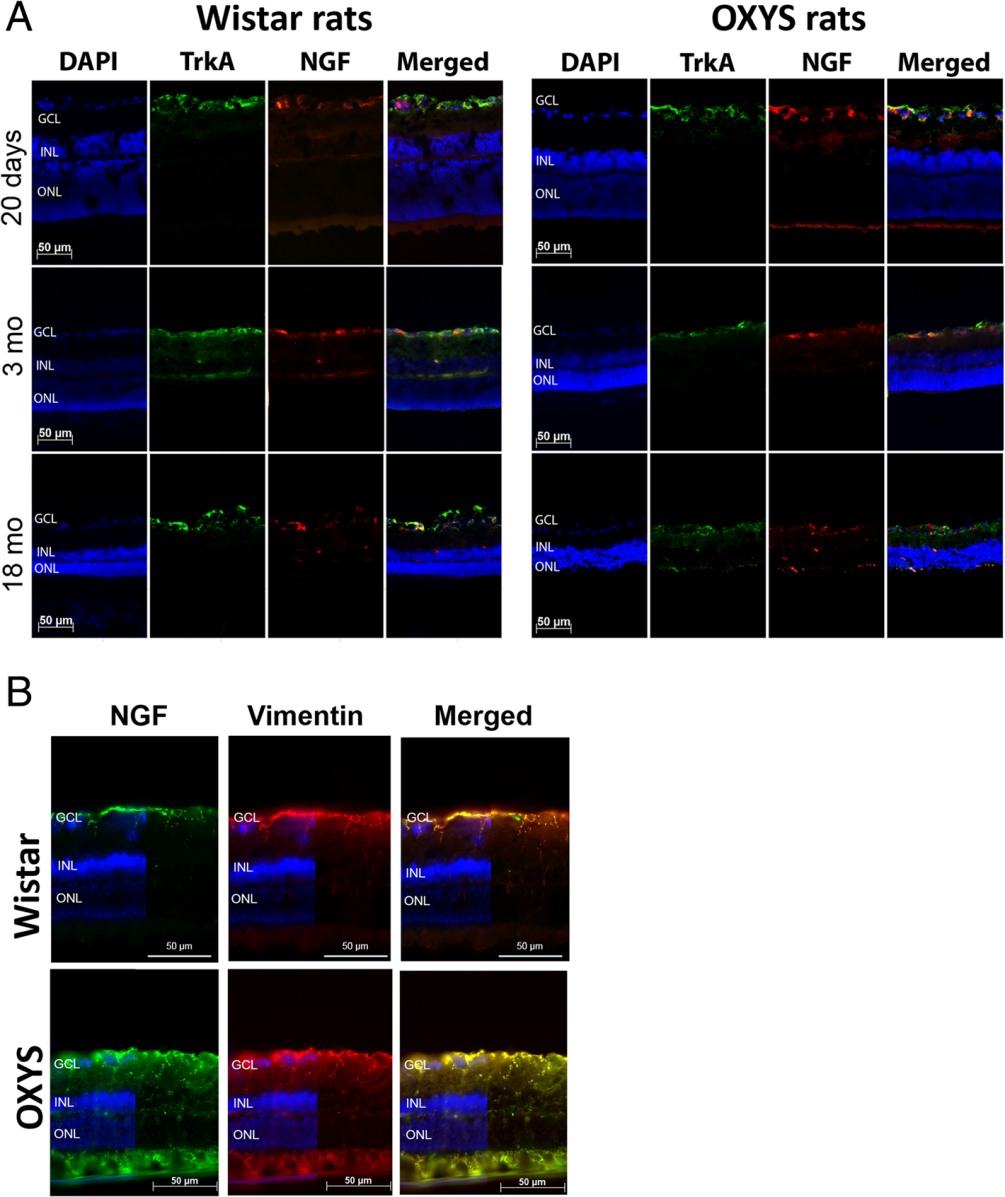
mNGF and TrkA expression in the rat retina during aging and development of AMD-like retinopathy. **a** Double-label immunofluorescent staining of mNGF (red) and TrkA (green) in the retina of OXYS and Wistar rats (*n* = 4) at various ages. **b** Double-label immunofluorescent staining of vimentin (red) and mNGF (green) in the retina of 18-month-old OXYS and Wistar rats. Vimentin expression increased at the advanced stage of AMD-like retinopathy. There was good colocalization of vimentin and mNGF in the OXYS retina. Nuclei were stained blue with DAPI. Abbreviations: GCL, ganglion cell layer; INL, inner nuclear layer; ONL, outer nuclear layer

In the retina, mNGF and its receptor TrkA support the survival of retinal ganglion cells and photoreceptors, maintaining the development and homeostasis of the retina. To understand whether NGF is affected in OXYS and Wistar rats’ retina at different ages, serial histological sections were probed with a specific anti-NGF antibody and anti-TrkA antibody. Analysis of the results of immunohistochemical staining of the retinal slices showed that NGF and TrkA proteins was mostly concentrated in the ganglion cell layer (GCL) of rats of both strains and all ages. We did not detect changes in the expression of proteins NGF and TrkA between OXYS and Wistar rats at age 20 days or 3 months. NGF and TrkA staining was observed in the GCL and inner nuclear retinal layer (INL) of 3-month-old Wistar rats, while in OXYS rats, proteins NGF and TrkA localized in the GCL at this age. Colocalization of proteins NGF and TrkA in the retina of Wistar rats increased by age 3 months in the GCL and became greater than that in OXYS rats. The amounts of NGF and TrkA in the retina of OXYS and Wistar rats increased by age 18 months and became higher than those in 3-month-old rats of both strains. By the age of 18 months, the amounts of NGF and TrkA increased in the retina of the OXYS strain, and these proteins were detectable in all layers of the retina except for the photoreceptor layer and RPE. On the contrary, in the Wistar retina, the staining of NGF was detectable in the GCL and TrkA expression was detectable in the GCL and INL, but colocalization of proteins NGF and TrkA decreased as compared to age 3 months and age-matched OXYS rats (Fig. 1a).

To identify cell type–specific expression of NGF, we used a double-labeling procedure with antibodies against vimentin (Muller cell maker). Vimentin and NGF expression patterns were often colocalized in the cells of the GCL and in Muller processes across the plexiform layers in OXYS rats at the age of 18 months, suggesting that glial cells accumulate NGF during progressive stages of retinopathy (Fig. 1b).

### mBDNF and TrkB expression

The roles of BDNF in retinal pathology, neuroprotection, and oxidative stress have extensively been studied over the past years [15]. Nevertheless, most of this research has focused on the involvement of BDNF–TrkB signaling in tension glaucoma, primary open angle glaucoma, and diabetic retinopathy, but the alterations in both proBDNF and mBDNF expression levels and patterns during the development of AMD are barely known.

**Fig. 2:**
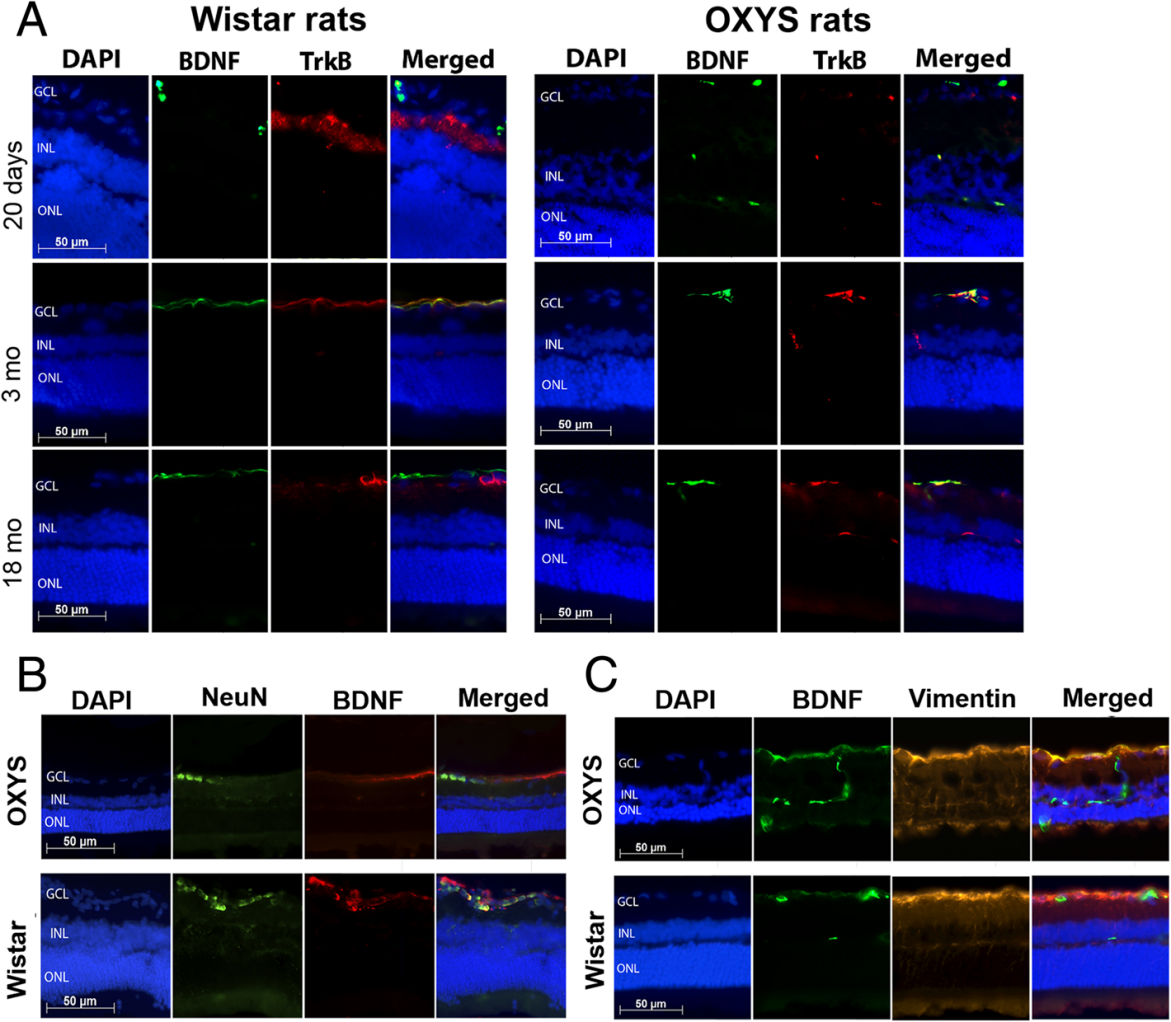
mBDNF and TrkB expression in the rat retina during aging and development of AMD-like retinopathy. **a** Double-label immunofluorescent staining of mBDNF (green) and TrkB (red) in the retina of OXYS and Wistar rats (*n* = 4) at various ages. **b** Double-label immunofluorescent staining of NeuN (green) and mBDNF (red) in the retina of 3-month-old OXYS and Wistar rats. There was no mBDNF expression in the retinal ganglion cells of OXYS rats. **c** Double-label immunofluorescent staining of vimentin (red) and mBDNF (green) in the retina of 3-month-old OXYS and Wistar rats. There was mBDNF expression in the Muller glia of both OXYS and Wistar rat retinas. Nuclei were stained blue using DAPI. Abbreviations: GCL, ganglion cell layer; INL, inner nuclear layer; ONL, outer nuclear layer

To assess the function of mBDNF and TrkB during rat retinal aging and development of retinopathy, we performed double-label immunofluorescence. mBDNF staining was observed in the GCL, a TrkB signal was detected in the inner plexiform layer of 20-day-old Wistar rats, whereas in OXYS rats, the mBDNF protein was detected in the GCL and INL, but TrkB in GCL at this age. No colocalization of proteins mBDNF and TrkB was observed in the retina of OXYS and Wistar strains. The amounts of mBDNF and TrkB and their colocalization in the retina of Wistar rats increased by age 3 months and became greater than those in OXYS rats. Immunofluorescence of mBDNF was concentrated in the GCL, whereas TrkB signals were detected in the GCL and INL of rats of both strains at the ages of 3 and 18 months. Colocalization of proteins mBDNF and TrkB increased in the retina of 3-month-old Wistar rats as compared to age-matched OXYS rats. At the age of 18 months, we noticed an insignificant reduction of mBDNF and TrkB labeling in the retina of rats of both strains. We did not observe differences in the expression and colocalization of proteins mBDNF and TrkB between OXYS and Wistar rats at this age (Fig. 2a).

Even though we observed only subtle changes in the mBDNF and TrkB staining during the development of AMD-like retinopathy in OXYS rats, we saw a difference in the cell type–specific expression of mBDNF between OXYS and Wistar rats. Using antibodies against vimentin (Muller cell maker) and NeuN (ganglion cell marker), we demonstrated that mBDNF was expressed specifically in Muller cells in OXYS rats’ retina, whereas mBDNF immunoreactivity was detected as retinal ganglion cells (positively stained) and Muller cells in the retina of Wistar rats (Fig. 2b, c). These data indicated that mBDNF was localized in astrocytes and Muller cells during the development of AMD-like retinopathy in OXYS rats.

### proBDNF and p75^NTR^ expression

**Fig. 3:**
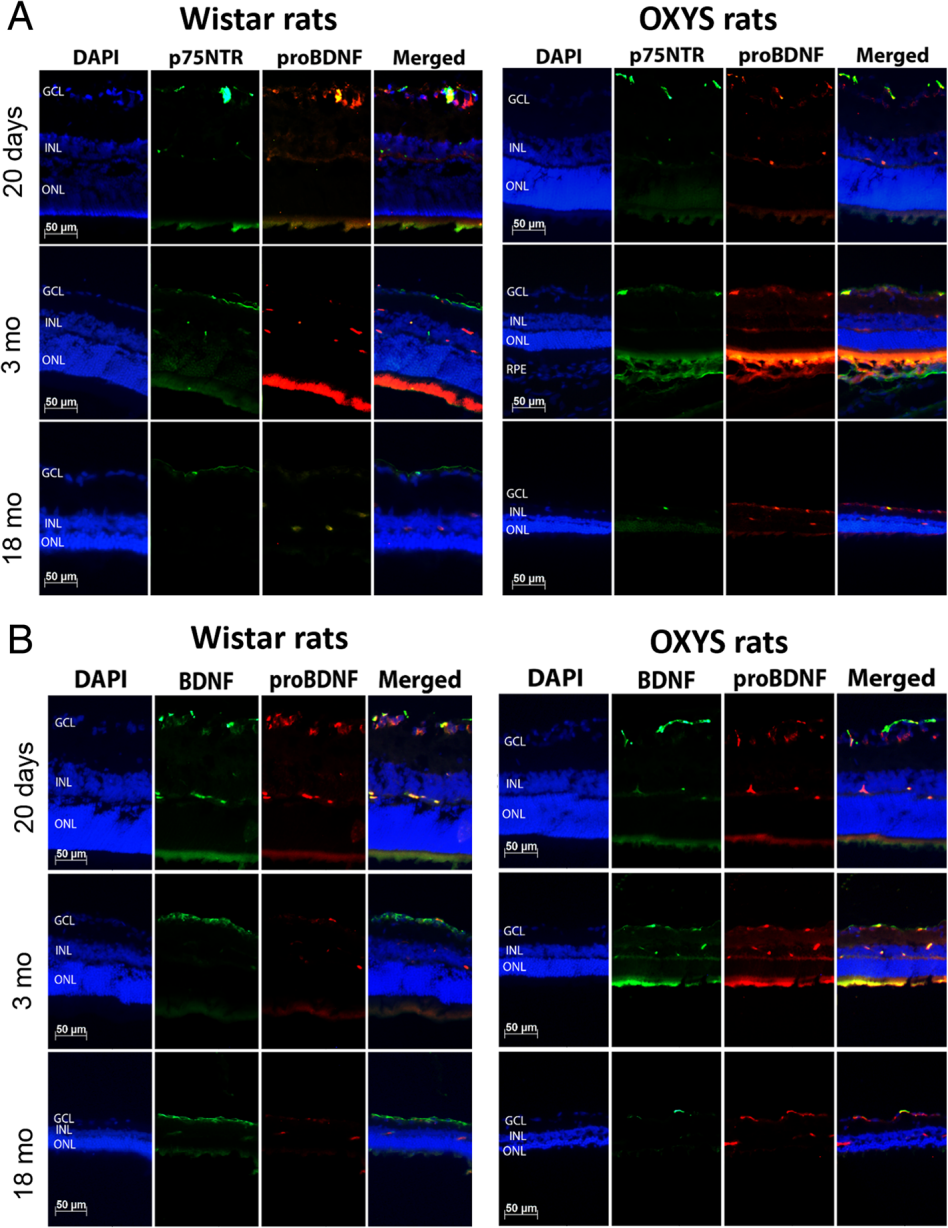
proBDNF, mBDNF and p75NTR expression in the rat retina during aging and development of AMD-like retinopathy. **a** Double-label immunofluorescent staining of proBDNF (red) and p75NTR (green) in the retina of OXYS and Wistar rats (*n* = 4) at various ages. **b** Cryosections of OXYS and Wistar rat retinas were stained with antibodies against proBDNF (red) and mBDNF (green). proBDNF dominated over mBDNF in OXYS rats at all ages in comparison with Wistar rats. Abbreviations: GCL, ganglion cell layer; INL, inner nuclear layer; ONL, outer nuclear layer

At the next step, we investigated the immunolabeling of proBDNF and its receptor p75^NTR^ in the retina of OXYS and Wistar rats during the development of AMD-like retinopathy. The immunostaining of retinal cryosections revealed that proBDNF and p75^NTR^ were present only in the GCL, INL, and outer plexiform layer (OPL). We did not detect differences in the labeling of proteins proBDNF and p75^NTR^ between OXYS and Wistar rats at age 20 days, but colocalization of these proteins in OXYS rats was greater than that in Wistar rats. This result may mean an increase of cell death in the 20-day-old OXYS retina. Indeed, an increase in apoptosis has been observed in OXYS rats at age 20 days as compared age-matched Wistar rats [9]. With age, the amount of proBDNF and p75^NTR^ and their colocalization decreased in Wistar rats. By the age of 3 months in OXYS rats in contrast to Wistar rats, proBDNF labeling increased and was observed in the GCL, INL, and OPL. By age 18 months, the proBDNF labeling increased in the retina of the OXYS strain, and colocalization of proBDNF with its receptor p75^NTR^ increased in the GCL. Notably, the observed an increase in the colocalization of proBDNF and p75^NTR^ in the retina of old OXYS rats, and this alteration was accompanied by a reduction in the number of the photoreceptor cells in the outer nuclear layer and in the number of ganglion neurons in the GCL (Fig. 3a). Our recent studies point to a modest role of apoptosis at advanced stages of retinopathy in OXYS rats [9] and preferential involvement of other types of cell death (such as autophagy and necroptosis) in the retina during AMD [14].

To better understand the biological significance of the upregulation of proBDNF found in OXYS rats’ retina, we also determined the localization of proBDNF and mBDNF in samples of different stages of AMD-like retinopathy and in the control animals. We found greater protein labeling of proBDNF relative to mBDNF in 20-day-old rats of both strains. With age, the immunostaining of mBDNF increased and that of proBDNF decreased in Wistar rats. At the age of 18 months, we detected only a faint immunofluorescent signal of proBDNF. In contrast to Wistar rats, retinal proBDNF was upregulated and its amount exceeded that of mBDNF in OXYS rats at ages 3 and 18 months (Fig. 3b).

## Discussion

Here we revealed alterations of the expression and localization of neurotrophic factors in the retina of senescence-accelerated OXYS rats, which spontaneously develop a retinopathy similar to AMD. Overall, our results indicate disturbances in the neurotrophic support in the retina of OXYS rats: 1) we found increased NGF staining in Muller cells during the progressive stage of AMD-like retinopathy; 2) the mBDNF protein localized in Muller cells in OXYS rats, whereas mBDNF immunoreactivity was detected as retinal ganglion cells (positively stained) and Muller cells in the retina of Wistar rats; 3) during the development of AMD-like retinopathy in OXYS rats, proBDNF dominated over mBDNF thus increasing cell loss in the retina.

NGF supports the survival of neurons and reduces degeneration directly or induces expression of other neurotrophic factors in Müller cells to indirectly promote photoreceptor survival. The expression of mNGF and its high-affinity receptor TrkA is increased in a compensatory manner in many pathological conditions to protect tissue function, for example, in ocular hypertension, neurogenic inflammation, and oxygen-induced retinopathy [4]. Thus, the normally low basal production of NGF is enormously upregulated during an inflammatory response and because of tissue damage. A number of studies have shown that cytokines involved in inflammation are promoters of NGF synthesis in a variety of cell types [16]. In the present study, we report that NGF staining increases in Muller cells in 18-month-old OXYS rats as compared to the age-matched Wistar rats (and to the previous stage in OXYS rats). Previously, our group demonstrated that during active progression of the disease–ages 7 and 18 months, when most OXYS rats show alterations similar to third-stage AMD in humans—reactive gliosis takes place [10]. Gliosis of Müller cells has both cytoprotective and cytotoxic effects on retinal neurons [17]. NGF prevents the osmotic swelling of Müller cells by inducing autocrine and/or paracrine FGF signaling and indirectly controls the swelling of bipolar cells by inducing a release of cytokines from Müller cells [18]. On the other hand, some data suggest that NGF has the potential to induce VEGF production and increase Müller cell proliferation indicating that an excessive dose of NGF might induce gliosis, which might lead to secondary injury [19]. VEGF is neuroprotective at low concentrations, but at high concentrations, it causes all sorts of vascular pathologies. Previously, we showed that the development of retinopathy in OXYS rats takes place at reduced concentrations of VEGF and PEDF [20, 21]. We suggested that retinal degeneration in OXYS rats progresses even in the presence of compensatory upregulation of NGF. Our present data indicate that the increase in endogenous NGF levels in Muller cells is not sufficient to counteract neurotoxic mechanisms and to support the survival of the neuron and photoreceptors, and that other events (probably affecting NGF receptor expression) might contribute to neurodegeneration.

The retina is an extension of the CNS, and BDNF is reported to have potential neuroprotective effects in some neurodegenerative ocular disorders [15]. BDNF is expressed in different types of neurons (e.g., ganglion and amacrine neurons) and glia (e.g., astrocytes, Muller cells), but the localization of BDNF in photoreceptors is controversial [22, 23]. Our study revealed the expression of proBDNF, mBDNF, and its receptors (p75NTR and TrkB) in the GCL, INL, and OPL. Thus, we did not detect BDNF in photoreceptor cells in normal and AMD-like retina by immunostaining.

In the retina, the neurotrophic effect of BDNF is in part mediated by a direct action on retinal neurons [24], and by an indirect mechanism involving Müller cells [25]. Nevertheless, it was shown that TrkB signaling in Müller cells, but not in retinal ganglion and amacrine cells, is deeply involved in neural protection and regeneration during retinal degeneration [26] and that the BDNF-induced protection of photoreceptors from light damage is mediated by activation of truncated TrkB expressed by Müller cells [27]. This hypothetical model is consistent with our results showing the amounts of mBDNF protein in Muller cells in OXYS rats compared to Wistar rats. In addition, BDNF usually undergoes retrograde transport from the tectum to the retina and is synthesized within the retina [28]. BDNF production in the retina may serve as an endogenous neuroprotective system in response to pathological conditions. The BDNF protein content of Muller cells in OXYS rats can also point to obstructed BDNF transport in the optic nerve during the development of retinopathy.

Our results indicate that during the development of AMD-like retinopathy in OXYS rats, proBDNF dominated over mBDNF, thereby leading to increased cell loss in retina. Secretion and processing of proBDNF and/or BDNF might be involved here too. It was suggested that neurons have a limited ability to process proBDNF [29].

## Conclusions

Although many researchers reported that topical NGF and/or BDNF treatment of eyes may be a potential effective therapy for AMD [15, 30], it still seems difficult to bring this insight to the clinic. Greater amounts of empirical data in translational and clinical settings need to be pooled to reproduce these findings in a real-life setting. Our data indicate that the wide spectrum of age-related retinal alterations in humans and differences in the risk of AMD may be attributed to genetic differences and dissimilarity in immune-system factors [31].

Taken together, these data indicate that alterations in the balance of neurotrophic factors in the retina are involved in the development of AMD-like retinopathy in OXYS rats and confirm their participation in the pathogenesis of AMD in humans. This study adds to the growing recognition of the importance of Muller cells in the maintenance and support of other retinal cell types by producing neurotrophins or facilitating their trafficking.

## Abbreviations

AMD: Age-related macular degeneration
BDNF: Brain-derived neurotrophic factor
GCL: Ganglion cell layer
INL: Inner nuclear layer
NGF: Nerve growth factor
ONL: Outer nuclear layer
OPL: Outer plexiform layer
RPE: Retinal pigment epithelium
TrKA: Tropomyosin-receptor kinase A
TrkB: Tropomyosin-receptor kinase B
